# Supplementary material for: Nearest-Neighbour and Non-Nearest-Neighbour Non-Covalent Interactions between Substituents in the Aromatic Systems: Experimental and Theoretical Investigation of Functionally Substituted Benzophenones
Source: Molecules. 2022 Dec 2;27(23):8477. doi: 10.3390/molecules27238477 (PMC9738408; doi:10.3390/molecules27238477)
Supplement: Supplementary file 1 [file molecules-27-08477-s001.zip › molecules-2036463-supplementary.pdf]

Electronic supporting information

Nearest-neighbour and non-nearest-neighbour non-covalent interactions between substituents in the aromatic systems: Experimental and theoretical investigation of functionally substituted benzophenones

Artemiy A. Samarov,[a,*] Stanislav O. Kondratev,[b] Sergey P. Verevkin[b,c,*]

[a] *Saint Petersburg State University, Universitetsky pr. 26, Peterhof, Saint Petersburg, 198504, Russia.*

[b] *Department of Physical Chemistry and Faculty of Interdisciplinary Research, Competence Centre CALOR, University of Rostock, 18059 Rostock, Germany*

[c] *Department of Physical Chemistry, Kazan Federal University, 420008 Kazan, Russia*

Transpiration method

Absolute vapour pressures were measured using the transpiration method [1,2]. The main idea of this method is to saturate the gas stream flowing over the sample and to determine the amount of compound transferred by the gas within a given time. Approximately 0.5 to 0.8 g of the sample is mixed with glass beads (diameter 1 mm) and placed in the thermostatted U-shaped saturator. The glass beads are needed to enlarge the contact area between gas and sample. A stream of nitrogen at a well-defined flow rate was passed through the saturator at constant temperature (± 0.1 K), and the transported material was collected in a cold trap. The amount of the condensed substance was determined by GC. The saturation vapour pressure p_i at each temperature T_i was calculated from the amount of condensate collected within a definite period of time:

$$p_i = m_i \cdot R \cdot T_a / V \cdot M_i \; ; \qquad V = (n_{N2} + n_i) \cdot R \cdot T_a / P_a \qquad \text{(S1)}$$

where V is the volume of the gas phase consisting of the n_{N2} moles of the carrier gas and n_i mole of gaseous compound under study (with the molar mass M_i) at the atmospheric pressure P_a and the ambient temperature T_a. The volume of the carrier gas V_{N2} was determined by the digital flow rate sensor from integration with a microcontroller. We used the Honeywell S&C - HAFBLF0200C2AX5 digital flow rate sensor with uncertainty at the level of 2.5 %. The flow rate of the nitrogen stream was also controlled by using a soap bubble flow meter (HP soap film flowmeter (model 0101-0113)) and optimized in order to reach the saturation equilibrium of the transporting gas at each temperature under study. The volume of the carrier gas V_{N2} was readied from the digital flow rate sensor. The amount of the compound under investigation n_i in the carrier gas was estimated at each temperature using the ideal gas law.

Before starting the vapour pressure measurements, the sample was first pre-conditioned at 310-320 K (within about one hour) in order to remove possible traces of water. The saturator was then kept at 310-315 K (to remove possible traces of volatile compounds). In order to assure the competition of pre-conditioning at the selected temperature, three samples were taken during the sample flashing at and analysed by the GC. A constant vapour pressure at this temperature indicated that the transpiration experiments could begin. GC analysis of the transported material did not reveal any additional contamination. The absence of impurities and decomposition products was re-checked by GC analysis of the saturator content at the end of the entire series of experiments.

Knudsen effusion method [3]

Vapor pressures of 2,4-di-hydroxy-benzophenone and 2,2´,4,4´-di-hydroxy-benzophenone were measured using the Knudsen effusion methods. This method is used to determine the vapor pressure at constant temperature of a single compound, from the measurement of the weight loss through a small orifice into a vacuum. The Knudsen effusion cell consists, essentially, of a cell having a small orifice of known diameter and immersed in a container connected to a high-vacuum system. The cell is weighed at the beginning of the experiment and at time intervals with a balance, which can be external or internal to the apparatus. Alternatively, the vapor can be condensed on a liquid nitrogen cooled cold finger or on a surface cooled by dry ice-acetone or liquid nitrogen placed above the orifice, removed, and analysed.

The constant temperature in the measuring cell was maintained within the brass heating block with the stability of 0.02 K in the interval (300 to 470) K provided by a Watlow EZ-ZONE PM EXPRESS PM6C1CA-AAAAAAA controller equipped with a 3-wires Pt 100 Ohm resistance thermometer. The temperature in the heating block was measured using a precise platinum resistance thermometer Pt100 GTF 401 1/10 DIN (-50 ... +400°C, 1/10 DIN Class B (±0.03°C at 0°C) Greisinger GMH 3710 with 4-wire connection calibrated according to DIN EN 60751 by the German Calibration Service (DKD).

The chamber is wrapped with an insulation based on polyamide band Kapton and SLENTEX mineral insulation. This prevents the chamber from temperature fluctuations related to heat exchange with the environment. The block is attached to the vacuum chamber with the aid of a blank steel flange (ISO100BK-304 with centring ring) with an orifice for the heating chamber. The vacuum chamber is a T-shaped vacuum tube. It is connected to the pumping system via gate valve and joined with a cold trap. The cold trap has purpose of catching effused substance in one place to avoid vacuum chamber contamination. It is a stainless-steel cylinder with a closed bottom that contains two consequent Peltier elements that create low temperature for the trap that allow to condensate the effused vapour. The Peltier elements in turn are cooled with a plate heat exchanger Watercool HEATKILLER® NSB Rev3.0 Nickel connected to a cold-water source.

The pumping system includes piping and a pump Agilent Technologies DS202 that is providing vacuum creation of 10^{-5} Pa. The depth of vacuum is measured with Full Range Pirani Bayard-Alpert Gauge Agilent Technologies FRG-720. Effusion cell is a two-part cylinder where first part is a bottom that is supposed to contain analysed substance and the second part is a lid with an orifice. The bottom part is an open top cylinder. It is made of stainless steel 316Ti and has diameter of 25 mm and the height of 10 mm. The lid can contain a golden round membrane with an orifice of either 0.5 mm, 1 mm, 1.4 mm, or 2 mm diameters. The orifices were made with the aid of drilling machine of corresponding sizes.

The vapour pressure at the temperature of the cell T is usually calculated with Eq. S2

$$p^{*} = \frac{\Delta m}{kAt} \cdot \sqrt{\frac{2\pi RT}{M}} \qquad \text{(S2)}$$

where t is the vacuum exposure time, M – molar mass of vapour, Δm – mass loss, A – orifice area, R – molar gas constant, and k – transmission probability factor. The factor k is a complex factor that is a function of membrane thickness l, orifice radius r, and Knudsen number Kn and considers effusing gas isotropy failure according to Wahlbeck [4].

In first approximation it is assumed that the vapour reaches full saturation. In fact, that is an unrealistic situation. The phenomenon of undersaturation was studied by Nesmeianov [5]. From his work it can be concluded that the undersaturation cannot be left unconsidered especially for solid substances. The reason for undersaturation is that the effusion rate is higher than the rate of sublimation itself. Zaitsau [6] in his work analysed the issue and transformed the equation (S3) into the following corrected form:

$$p_{\mathrm{sat}} = \frac{\Delta m}{kAt} \cdot \sqrt{\frac{2\pi RT}{M}} \cdot (1 + kA \cdot (\alpha\gamma A_{\mathrm{sub}})^{-1}) = p^{*} \cdot (1 + kA \cdot (\alpha\gamma A_{\mathrm{sub}})^{-1}) \quad \text{(S3)}$$

where α is the condensation coefficient, γ is the roughness coefficient and A_{sub} is ideal geometrical surface of the sample from which the sublimation occurs. The $\alpha\gamma A_{\mathrm{sub}}$ product is assumed to be temperature independent and was adjusted by least-squares fitting with Clausius-Clapeyron equation. The detailed description is given in reference [7]. The $\alpha\gamma A_{\mathrm{sub}}$ was found to be (2.9 ± 0.9) 10^{-6} m^2.

Table S1
Thermochemical data at T = 298.15 K for auxiliary reference compounds ($p°$ = 0.1 MPa in kJ·mol^{-1}).

	$\Delta_f H_m^o$ (cr or liq)	$\Delta_l^g H_m^o$	$\Delta_f H_m^o$(g)$_{\mathrm{exp.}}$
benzene (liq)	49.0±0.9 [8]	33.9±0.1 [8]	82.9±0.9 [8]
acetophenone (liq)	-142.5±1.0 [9]	55.4±0.3 [10]	-87.1±1.0
phenol (liq)		58.0±0.6 [11]	-92.5±1.2 [11]
methoxy-benzene	-116.9±0.7 [11]	46.4±0.2 [11]	-70.3±0.7 [11]
1,2-dimethoxy-benzene (liq) [12,13]	-270.5±3.1	64.5±0.3	-206.0±3.1
1,3-dimethoxy-benzene (liq) [12,13]	-283.3±1.9	59.7±0.2	-223.6±1.9
1,4-dimethoxy-benzene (cr) [12,13]	-296.5±0.9	80.3±0.2	-216.2±0.9
1,4-dimethoxy-benzene (liq) [12,13]	-	61.6±0.2	-

Benzophenone	Structure	$\Delta_f H_m^o(g)_{G4}$[a]/ kJ·mol^{-1}
2-hydroxy-benzophenone	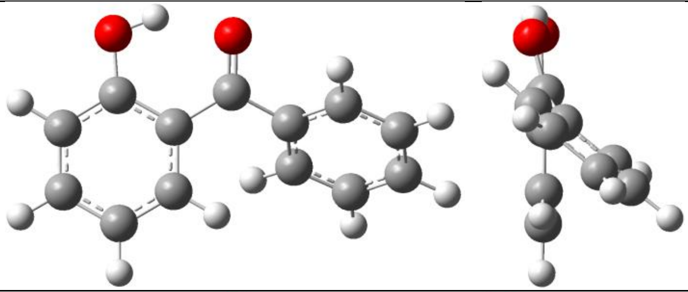	-149.9
3-hydroxy-benzophenone	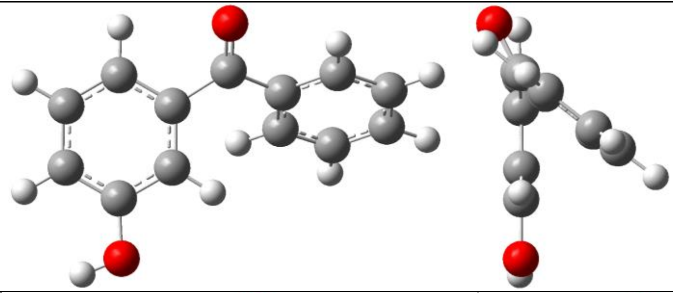	-124.5
4-hydroxy-benzophenone	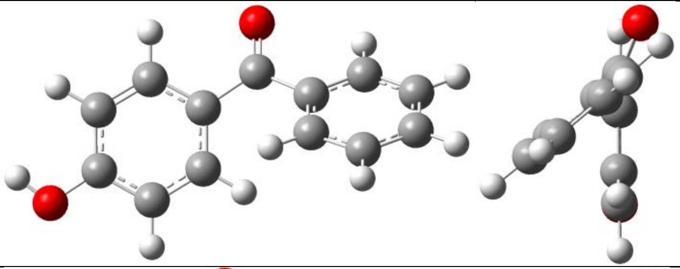	-128.8
2-methoxy-benzophenone	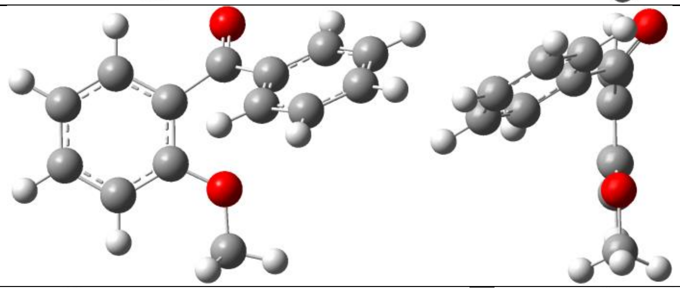	-101.2
3-methoxy-benzophenone	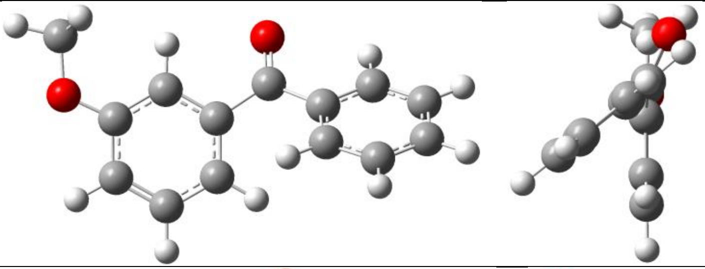	-107.3
4-methoxy-benzophenone	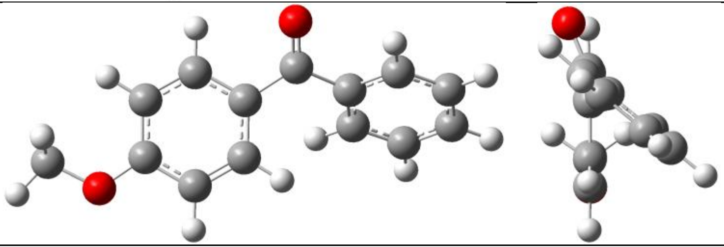	-108.5

[a] Calculated using the atomisation procedure.

Fig. S1. The stable conformers of hydroxy- and methoxy-substituted benzophenones.

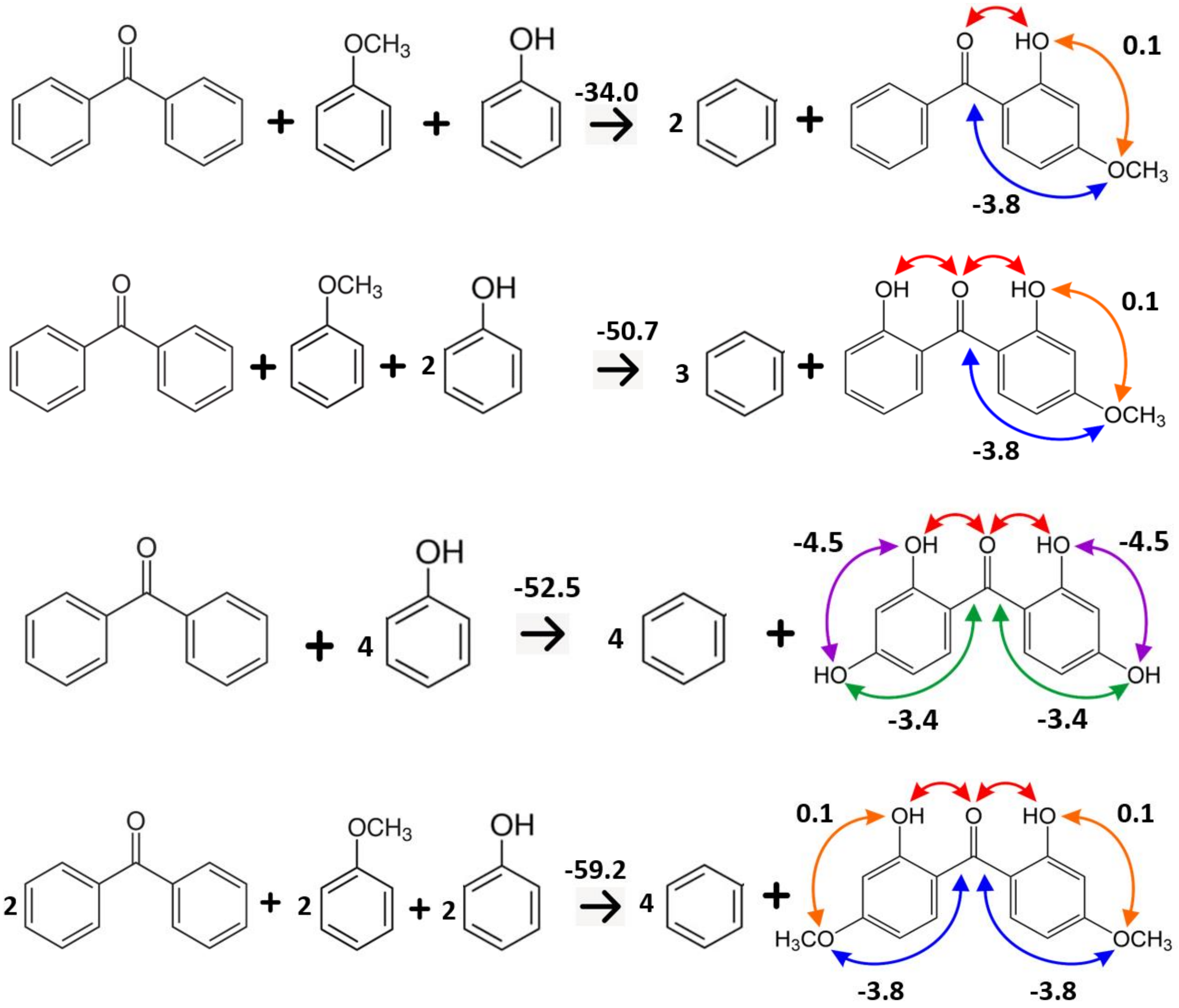

Fig. S2. Determination of the intra-molecular hydrogen bonding strength in substituted benzophenone using the "well-balanced reaction" method (All values are in kJ·mol^{-1})

Enthalpy of fusion measurements

Thermal behavior of crystalline sample of 2,4-dihydroxy-benzophenone including melting temperature, solid-solid phase transition and enthalpy of fusion was studied with a commercial PerkinElmer DSC Pyris 1 equipped with an intracooler. A sample was placed in the standard non-pinned aluminium pan of 40 μl volume. Pan and sample were weighted with a microbalance with the standard uncertainty of $5 \cdot 10^{-6}$ g. In the first DSC run the sample was heated with a rate 10 K·min^{-1} to 398 K (~ 30 K above melting temperature provided by supplier) and then cooled down to 298 K, also with the rate10 K·min^{-1}. Such procedure provided sufficient contact between the sample and the bottom of pan. The DSC experiments were repeated three times. The calibration of the DSC was checked with melting behaviour of reference indium sample. The twice standard deviation of the enthalpy of fusion in the test measurements for reference compound was ± 0.3 kJ·mol^{-1} and ± 0.3 K for the melting temperature. Uncertainties of the enthalpy of fusion values are expressed as expanded uncertainties (at a level of confidence of 0.95, k=2). They include uncertainties from fusion experiment and calibration. Details are reported elsewhere [14].

As a rule, thermochemical calculations are commonly performed at the reference temperature T = 298.15 K. The adjustment of $\Delta_{\mathrm{cr}}^{\mathrm{l}} H_{\mathrm{m}}^{\mathrm{o}}(T_{\mathrm{fus}})$ was performed with help of the equation [15]:

$\Delta_{cr}^{l}H_{m}^{o}$(298.15 K)/(J·mol^{-1}) = $\Delta_{cr}^{l}H_{m}^{o}$(T_{fus}/K) – ($\Delta_{cr}^{g}C_{p,m}^{o}$-$\Delta_{l}^{g}C_{p,m}^{o}$)×[($T_{fus}$/K) – 298.15 K] (S1)
where $\Delta_{cr}^{g}C_{p,m}^{o}$ and $\Delta_{l}^{g}C_{p,m}^{o}$ were taken from Table S2. With this adjustment, the molar enthalpies of fusion, $\Delta_{cr}^{l}H_{m}^{o}$(298.15 K) were calculated. Uncertainties in the temperature adjustment of fusion enthalpy from T_{fus} to the reference temperature were estimated to account with 30% to the total adjustment [16].

References

1. Kulikov, D.; Verevkin, S.P.; Heintz, A. Determination of Vapor Pressures and Vaporization Enthalpies of the Aliphatic Branched C 5 and C 6 Alcohols. *J. Chem. Eng. Data* **2001**, *46*, 1593–1600, doi:10.1021/je010187p.
2. Verevkin, S.P.; Emel'yanenko, V.N. Transpiration method: Vapor pressures and enthalpies of vaporization of some low-boiling esters. *Fluid Phase Equilib.* **2008**, *266*, 64–75, doi:10.1016/j.fluid.2008.02.001.
3. Verevkin, S.P. Chapter 1. Pure Component Phase Changes Liquid and Gas. In *Experimental Thermodynamics: Measurement of the thermodynamic properties of multiple phases. (Editors: R.D. Weir; Th.W. De Loos), Vol 7, Elsevier*; 2005; pp. 6–30.
4. Wahlbeck, P.G. Effusion. VII. The Failure of Isotropy of a Gas in an Effusion Cell and the Transition Region. *J. Chem. Phys.* **1971**, *55*, 1709–1715, doi:10.1063/1.1676300.
5. Nesmeianov, A.N.; Carasso, I.J. *Vapour pressure of the elements, Academic Press, New York*; 1963;
6. Zaitsau, D.; Kabo, G.J.; Kozyro, A.A.; Sevruk, V.M. The effect of the failure of isotropy of a gas in an effusion cell on the vapor pressure and enthalpy of sublimation for alkyl derivatives of carbamide. *Thermochim. Acta* **2003**, *406*, 17–28, doi:10.1016/S0040-6031(03)00231-4.
7. Bazyleva, A.B.; Blokhin, A.V.; Kabo, G.J.; Kabo, A.G.; Sevruk, V.M. Thermodynamic properties of 2-adamantanone in the condensed and ideal gaseous states. *Thermochim. Acta* **2006**, *451*, 65–72, doi:10.1016/j.tca.2006.08.018.
8. Roux, M.V.; Temprado, M.; Chickos, J.S.; Nagano, Y. Critically evaluated thermochemical properties of polycyclic aromatic hydrocarbons. *J. Phys. Chem. Ref. Data* **2008**, *37*, 1855–1996, doi:10.1063/1.2955570.
9. Colomina, M.; Latorre, C.; Perez-Ossorio, R. Heats of combustion of five alkyl phenyl ketones. *Pure Appl. Chem.* **1961**, *2*, 133–136, doi:10.1351/pac196102010133.
10. Steele, W. V; Chirico, R.D.; Knipmeyer, S.E.; Nguyen, A. Vapor Pressure of Acetophenone, (±)-1,2-Butanediol, (±)-1,3-Butanediol, Diethylene Glycol Monopropyl Ether, 1,3-Dimethyladamantane, 2-Ethoxyethyl Acetate, Ethyl Octyl Sulfide, and Pentyl Acetate. *J. Chem. Eng. Data* **1996**, *41*, 1255–1268, doi:10.1021/je9601117.
11. Verevkin, S.P. Weaving a web of reliable thermochemistry around lignin building blocks: phenol, benzaldehyde, and anisole. *J. Therm. Anal. Calorim.* **2022**, *147*, 6073–6085, doi:10.1007/s10973-021-10924-x.
12. Varfolomeev, M.A.; Abaidullina, D.I.; Solomonov, B.N.; Verevkin, S.P.; Emel'yanenko, V.N. Pairwise Substitution Effects, Inter- and Intramolecular Hydrogen Bonds in Methoxyphenols and Dimethoxybenzenes. Thermochemistry, Calorimetry, and First-Principles Calculations. *J. Phys. Chem. B* **2010**, *114*, 16503–16516, doi:10.1021/jp108459r.
13. Verevkin, S.P.; Konnova, M.E.; Turovtsev, V. V.; Riabchunova, A. V.; Pimerzin, A.A. Weaving a Network of Reliable Thermochemistry around Lignin Building Blocks: Methoxy-Phenols and Methoxy-Benzaldehydes. *Ind. Eng. Chem. Res.* **2020**, *59*, 22626–22639, doi:10.1021/acs.iecr.0c04281.
14. Emel'yanenko, V.N.; Zaitsau, D.H.; Shoifet, E.; Meurer, F.; Verevkin, S.P.; Schick, C.;

Held, C. Benchmark Thermochemistry for Biologically Relevant Adenine and Cytosine. A Combined Experimental and Theoretical Study. *J. Phys. Chem. A* **2015**, *119*, 9680–9691, doi:10.1021/acs.jpca.5b04753.
15. Chickos, J.S.; Acree, W.E. Enthalpies of Vaporization of Organic and Organometallic Compounds, 1880–2002. *J. Phys. Chem. Ref. Data* **2003**, *32*, 519–878, doi:10.1063/1.1529214.
16. Gobble, C.; Chickos, J.; Verevkin, S.P. Vapor Pressures and Vaporization Enthalpies of a Series of Dialkyl Phthalates by Correlation Gas Chromatography. *J. Chem. Eng. Data* **2014**, *59*, 1353–1365, doi:10.1021/je500110d.
